# Deficiency of Acetyl-CoA Carboxylase Impairs Digestion, Lipid Synthesis, and Reproduction in the Kissing Bug *Rhodnius prolixus*

**DOI:** 10.3389/fphys.2022.934667

**Published:** 2022-07-22

**Authors:** Bruno Moraes, Valdir Braz, Samara Santos-Araujo, Isadora A. Oliveira, Larissa Bomfim, Isabela Ramos, Katia C. Gondim

**Affiliations:** ^1^ Instituto de Bioquímica Médica Leopoldo de Meis, Universidade Federal do Rio de Janeiro, Rio de Janeiro, Brazil; ^2^ Centro de Espectrometria de Massas de Biomoléculas, Instituto de Biofísica Carlos Chagas Filho, Universidade Federal do Rio de Janeiro, Rio de Janeiro, Brazil

**Keywords:** *de novo* lipogenesis, oogenesis, fat body, *Rhodnius prolixus*, acetyl-CoA carboxylase (ACC)

## Abstract

*Rhodnius prolixus* is a hematophagous insect, vector of Chagas disease. After feeding, as blood is slowly digested, amino acids are used as substrates to fuel lipid synthesis, and adult females accumulate lipids in the fat body and produce eggs. In order to evaluate the importance of *de novo* fatty acid synthesis for this insect metabolism, we generated acetyl-CoA carboxylase (ACC) deficient insects. The knockdown (AccKD) females had delayed blood digestion and a shorter lifespan. Their fat bodies showed reduced *de novo* lipogenesis activity, did not accumulate triacylglycerol during the days after blood meal, and had smaller lipid droplets. At 10 days after feeding, there was a general decrease in the amounts of neutral lipids and phospholipids in the fat body. In the hemolymph, no difference was observed in lipid composition at 5 days after blood meal, but at day ten, there was an increase in hydrocarbon content and a decrease in phospholipids. Total protein concentration and amino acid composition were not affected. The AccKD females laid 60% fewer eggs than the control ones, and only 7% hatched (89% for control), although their total protein and triacylglycerol contents were not different. Scanning electron microscopy of the egg surface showed that chorion (eggshell) from the eggs laid by the AccKD insects had an altered ultrastructural pattern when compared to control ones. These results show that ACC has a central role in *R. prolixus* nutrient homeostasis, and its appropriate activity is important to digestion, lipid synthesis and storage, and reproductive success.

## Introduction

Hematophagous insects are vectors of various diseases—such as malaria, dengue fever, Chagas disease, and leishmaniosis—that affect millions of people over the world. *Rhodnius prolixus* is a hemipteran, member of the subfamily Triatominae that includes insects commonly known as kissing bugs. It is a vector of *Trypanosoma cruzi*, etiological agent of Chagas disease (also known as American trypanosomiasis), which is estimated to affect six to seven million people worldwide (https://www.who.int/health-topics/chagas-disease, April 2022). *R. prolixus* is an important vector of Chagas disease in South and Central Americas (de Fuentes-Vicente et al., 2018), and both nymphs and adult insects feed exclusively on blood, which is a very specific and limited diet. In hematophagous insects, feeding usually occurs in huge amounts and low frequency, triggers important events, as oogenesis and molting, and regulates the expression of diverse genes that take part in these processes. In this way, hematophagy is a very particular condition that results in specific metabolic adaptations.

In *R. prolixus*, after each blood meal, as digestion slowly occurs, lipids are absorbed by the enterocytes, metabolized, and secreted to the hemolymph, where they are transported by lipophorin (Lp), a major lipoprotein (Grillo et al., 2007). In the meantime, the fat body accumulates triacylglycerol (TG) in lipid droplets (LD), and oocytes develop in the vitellogenic ovaries (Pontes et al., 2008; Santos et al., 2011; Gondim et al., 2018; Silva-Oliveira et al., 2021). The ingested blood is a very limited nutrient source, as it is composed mainly of proteins, and carbohydrates and lipids are present in relatively low amounts (Lynch et al., 2017). Thus, hematophagous insects rely on amino acids derived from blood proteins to synthesize lipids (Gondim et al., 2018). Moreover, as these insects usually feed on large and infrequent meals, and it may take a long time before the next meal, lipid synthesis is finely regulated, and in *R. prolixus*, it is highly activated soon after feeding (Saraiva et al., 2021). In the *de novo* fatty acid synthesis pathway, the first step is catalyzed by the enzyme acetyl-CoA carboxylase (ACC), where acetyl-CoA is converted to malonyl-CoA that is a substrate for fatty acid synthase (FASN).

In insect females, ovaries accumulate proteins, glycogen, and lipids, which will constitute the yolk and will be used for embryo development (Gondim et al., 2018). Lipids are taken up from the hemolymph, as *de novo* lipogenesis in the oocytes seems to be minimal, and thus, oogenesis success depends on the acquisition of lipids from an exogenous source (Kawooya and Law, 1988; Ziegler, 1997). After emergence, *Aedes aegypti* adult females synthesize lipids from the sugar they feed and accumulate them in the fat body. These stores are mobilized after a blood meal, and lipids are then transported to the ovaries to sustain oocyte production (Ziegler and Ibrahim, 2001; Zhou et al., 2004). Similarly, in *Gryllus bimaculatus* females, the fat body synthesizes lipids *de novo* after adult emergence, to be used during vitellogenic oocyte growth (Lorenz and Anand, 2004).

Although hematophagous insects can *de novo* synthesize lipids, they can also obtain lipids directly from the ingested blood, and the contribution of each pathway is not known. In *Aedes aegypti* females, the knockdown of *ACC* or *FASN1* genes resulted in lower *de novo* lipid biosynthesis and reduced oviposition (Alabaster et al., 2011). However, the eggs laid by the ACC deficient females showed a defective eggshell, a phenotype that was not observed in eggs laid by females silenced for *FASN1*, indicating an effect unrelated to the inhibition of fatty acid synthesis itself. Moreover, the *FASN1* silenced insects had delayed blood meal digestion. The inhibition of *ACC* gene expression in the fat body of *Drosophila melanogaster* larvae caused a decrease in TG content and a concomitant increase in glycogen amounts (Parvy et al., 2012). Nonetheless, when ACC expression was inhibited in the oenocytes, the larvae did not complete development and died, and this phenotype was related to a defect in the water tightness of the spiracles, which control the entry of air into the trachea. This effect was possibly associated with a reduced synthesis of a putative very-long-chain-fatty acid within the oenocytes, due to a decrease in the activity of a specific elongase, as these enzymes also use malonyl-CoA, the product of ACC activity, as a substrate (Parvy et al., 2012). Thus, we may conclude that a decrease in ACC activity may affect not only parameters clearly related to lipid synthesis and energetic homeostasis, as lipid content, but it may also reveal some other unexpected relationships.

In *R. prolixus*, after feeding, Lp transports diacylglycerol, fatty acids, phospholipids, and cholesterol to the vitellogenic ovaries (Gondim et al., 1989; Santos et al., 2011; Entringer et al., 2021). These lipids are loaded onto circulating Lp at the midgut during blood digestion, but they may also be acquired from the fat body, where—except cholesterol—they may result from *de novo* lipogenesis (Saraiva et al., 2021). In this insect, lipid synthesis by the fat body is triggered by the blood meal, and amino acids released by the digestion of blood proteins are used as substrates (Saraiva et al., 2021). Unlike vertebrates, insects and all other arthropods have only one copy of the ACC gene (Saraiva et al., 2021). In the *R. prolixus* fat body, although expression levels of the ACC gene (*RhoprAcc*) and protein are higher in starving females, and decrease after feeding, *de novo* lipogenesis activity is stimulated by blood meal, in accordance with the TG accumulation that occurs after each feeding event, mediated by insulin signaling (Pontes et al., 2008; Silva-Oliveira et al., 2021). In order to evaluate the contribution of *de novo* lipogenesis to *R. prolixus* lipid utilization and metabolism, we have inhibited *RhoprAcc* (here referred to as *Acc*) gene expression and investigated possible effects on digestion, lipid synthesis and accumulation by the fat body, and reproduction.

## Material and Methods

### Insects

Experimental insects were adult mated females, fed in the outer ear of live rabbits at 3-week intervals. The colony of *Rhodnius prolixus* was maintained at 28 ± 2°C, relative humidity of 65%–85%, and 12 h/12 h light and dark cycles. All animal care and experimental protocols were conducted following the guidelines of the institutional animal care and use committee (Committee for Evaluation of Animal Use for Research from the Universidade Federal do Rio de Janeiro, CEUA-UFRJ), process number 01200.001568/2013-87, order number 149/19, and the NIH Guide for the Care and Use of Laboratory Animals (ISBN 0-309-05377-3).

### Quantitative PCR (qPCR)

Insects were dissected at the desired condition, and the obtained organs were washed in 0.15 M NaCl and homogenized in TRIzol reagent (Applied Biosystems, Inc., Foster City, United States), in pools of three organs. Total RNA was extracted according to the manufacturer’s protocol. Total RNA concentrations were measured by spectrophotometry with a Nanodrop ND-1000 (Thermo Fisher Scientific, Waltham, MA). The integrity and quality of the obtained total RNA samples were analyzed by electrophoresis on 1% agarose gel, and RNA was considered intact when the 18S rRNA band was observed. All samples showed an A260/A280 ratio between 1.9 and 2.0. RNA samples (1 µg) were treated with 1 U of DNase I (Thermo Fisher Scientific), and cDNA was synthesized with the High-Capacity cDNA Reverse Transcription Kit (Applied Biosystems) according to the manufacturer’s protocol. The qPCR reactions were performed in a StepOnePlus™ Real-Time PCR System thermocycler (Applied Biosystems) using the qPCRBIO SyGreen Mix Separate-ROX kit (PCR Biosystems Ltd, London, UK) and 3.0 pmol of each primer in 15 µL of final volume, as follows: 10 min at 95°C, followed by 40 cycles of 15 s at 95°C and 45 s at 60 °C. For the blanks, the cDNA was replaced by nuclease-free water. The Cq values obtained for the blanks were at least ten units higher than experimental points. The primers used in this study (Supplementary Table S1) were designed using Primer3 software (Rozen and Skaletsky, 2000) and were previously described (Saraiva et al., 2021). *Rhopr18S* gene amplification was used for normalization as described (Majerowicz et al., 2011), and its expression was constant under our experimental conditions, confirming that it was an appropriate and sufficient endogenous control (Bustin et al., 2009). The ΔΔCq values were calculated by the Cq values obtained as described (Livak and Schmittgen, 2001) and were used for statistical analyses. Relative expression values (2^−ΔΔCq^) were used only for data plotting.

### RNA Interference

Double stranded RNA (dsRNA) for the *Acc* (dsAcc) gene (VectorBase Gene ID: RPRC013987; Saraiva et al., 2021) was synthesized using the MEGAScript RNAi Kit (Thermo Scientific) and specific primers (Supplementary Table S1). dsRNA for the bacterial *MalE* gene (GenBank ID: 948,538) was used as control (dsMal) (Hansen et al., 2005). Unfed females were injected with 1 µg of dsRNA into the hemocoel, using a 10 µL microsyringe (Hamilton Company, Reno, NV, United States), and were fed 3 days later. This treatment generated the *Acc* knockdown (AccKD) females, and knockdown efficiency was confirmed four and 10 days after blood meal by qPCR as described above.

### Western Blots

Four days after blood meal, the females were dissected and the fat bodies and ovaries were homogenized (in pools of four) in 120 µL (fat bodies) or 150 µL (ovaries) of cold phosphate buffered saline (PBS; 10 mM sodium phosphate buffer, pH 7.4, 0.15 M NaCl), containing 1% protease inhibitor cocktail (Sigma-Aldrich, Saint Louis, United States). Samples were centrifuged at 14,000 *g* for 5 min, and the supernatants were subjected to Western blot, for the analysis of ACC protein expression, as described (Saraiva et al., 2021). The primary antibodies were rabbit monoclonal anti-human ACCβ (1:1,000 dilution; Cell Signaling Technologies, MA, United States; number C83B10), or mouse monoclonal anti-human ɑ tubulin (1:1,000 dilution; Santa Cruz Biotechnology, CA, United States; number (10D8): sc-53646). Horseradish-peroxidase conjugated goat anti-rabbit (1:20,000 dilution, Abcam; number ab6721) or goat anti-mouse (1:3,000 dilution; Abcam; number ab6789) were used as secondary antibodies, and the membranes were developed with an Enhanced Chemiluminescence (ECL) system (2.5 mM luminol, 0.4 mM coumaric acid, 0.02% hydrogen peroxide, 0.1 M Tris, pH 8.4), for 1 min.

### Protein Digestion Analysis

Total midguts (tissue and content) were obtained from dsRNA injected insects on days 1, 3, 5, and 10 after blood meal and stored at 70°C until use to follow digestion progress. Midguts were individually homogenized in 500 μL (final volume) of cold PBS, and the protein content of the samples was determined, using bovine serum albumin as standard (Lowry et al., 1951). Midguts were also dissected at first and 10th days after blood meal, individually homogenized in 500 µL of PBS, and total protein contents were analyzed by SDS-PAGE (12.5%).

Alternatively, females were fed on blood supplemented with heparin (5 U/ml; Hepamax-S, Blau Farmacêutica, São Paulo, Brazil) (Silva-Cardoso et al., 2018) in an artificial feeder (Garcia et al., 1975), and their body mass was measured at days 1 and 10 after feeding to determine weight loss resulting from blood digestion.

### Oviposition, Hatching, and Survival

For oviposition analysis, females were separated in individual vials immediately after feeding. The laid eggs were daily collected and counted until the end of the laying cycle, and hatching was also accompanied. Laid eggs were visually inspected and classified into two categories: 1) normal morphology, for those with shape and color similar to the control group; and 2) abnormal morphology, when any alteration was observed. For lifespan determination, these females were daily observed until all the insects had died.

### Protein and TG Quantification

Adult females were dissected at days 1, 3, 5, and 10 after blood meal to obtain fat bodies and ovaries, and at day 12 for chorionated oocytes. Laid eggs were obtained from females at days 7–10 after feeding, collected 0–24 h after oviposition. After washing, fat bodies and ovaries were individually homogenized in 200 µL of PBS; oocytes and eggs were homogenized (pools of 2–3) in 100 µL. Hemolymph was collected at days 5 and 10 after blood meal into a tube containing a few grains of phenylthiourea and centrifuged for 3 min at 6,000 *g*. Hemolymph supernatants and homogenates were stored at −20°C until use. The total protein content was determined as described above, and for TG quantification, the Triglicerides 120 colorimetric kit (Doles Reagents, Goiânia, Brazil) was used. Oocyte and egg homogenates were also subjected to an SDS-PAGE (7.5%) for the analysis of protein composition.

### Lipid Composition Analysis

Fat bodies from females at the fifth day after blood meal and hemolymph from days 5 and 10 were collected as described above and were subjected to lipid extraction in chloroform (Bligh and Dyer, 1959). The fat body neutral lipid and phospholipid composition was analyzed by thin-layer chromatography (TLC) on silica gel plates (Merck KGaA, Darmstadt, Germany) (Horwitz and Perlman, 1987; Kawooya and Law, 1988). The hemolymph neutral lipid composition was analyzed by high performance thin-layer chromatography (HPTLC) on silica gel plates (Merck) using two consecutive solvent systems (Fan et al., 2004). The plates were stained with copper reagent as described (Majerowicz et al., 2013), and the lipid relative composition was determined by densitometry using TotalLab Quant v11 (TotalLab Ltd., Newcastle, United Kingdom) with background corrections, after comparison with commercial lipid standards (Sigma Aldrich).

In order to evaluate hydrocarbon content in the eggshell surface, recently laid eggs were collected, emptied of yolk content with the help of tweezers, and washed in PBS. Groups of 20 eggshells were subjected to two consecutive extractions with 0.5 ml of hexane (Fan et al., 2008), extracted lipids were analyzed by TLC (Kawooya and Law, 1988), and the hydrocarbon spot was analyzed by densitometry, as described above.

### Scanning Electron Microscopy

For observation of the midgut luminal surface ultrastructure, anterior midguts were dissected from females at the 10th day after blood meal, washed in PBS, and longitudinally open. For the eggshell observation, chorionated (mature) oocytes were dissected and washed in PBS. Midguts and oocytes were fixed for 24 h in 2.5% glutaraldehyde (Grade I) and 4% freshly prepared formaldehyde in 0.1 M cacodylate buffer, pH 7.2. Samples were washed in cacodylate buffer, dehydrated in an ethanol series, critical point dried, and coated with a thin layer of gold. Samples were observed in a FEI Quanta 250 scanning electron microscope.

### Nile Red Staining of Lipid Droplets

Fat bodies were obtained from females on the fifth day after feeding and stained with Nile Red as described (Defferrari et al., 2016). Tissues were mounted in 100% glycerol and immediately imaged in a Leica TCS-SPE laser scanning confocal microscope (Leica Microsystems, Wetzlar, Germany). The average diameters of the lipid droplets were determined from four to five representative images obtained from four insects in each group, in three experiments, using the DAIME image analysis software after edge detection automatic segmentation (Daims et al., 2006), and the lipid droplets’ diameters were plotted in a frequency histogram (bin width = 1).

### *De novo* Lipid Synthesis Assay

Three days after blood meal, adult females were carefully dissected, midgut and ovary were removed, and the ventral fat body was kept intact, associated with the abdominal cuticle, as previously described (Atella et al., 1992). For the determination of the *de novo* lipid synthesis activity, fat bodies were incubated in the presence of ^3^H-acetate (0.1 μCi/μL; PerkinElmer, Waltham, MA, United States) in culture medium 199 (Sigma-Aldrich; catalog number M0393), supplemented with 10 mM sodium acetate, as described (Saraiva et al., 2021). After incubation, fat bodies were gently separated from the cuticle, washed in PBS, individually homogenized in 200 µL of PBS, and subjected to lipid extraction (Bligh and Dyer, 1959). Total radioactivity incorporated in lipids was determined by liquid scintillation counting.

### Hemolymph Amino Acid Composition

Five and 10 days after blood meal, hemolymph was collected from individual females, subjected to an extraction in chloroform (Bligh and Dyer, 1959), and the upper aqueous phase containing the water soluble molecules was separated. Samples were vacuum-dried, resuspended in 40 µL 70% acetonitrile (in water), and subjected (10 µL) to analysis by LC-MS on a Nexera X2 HPLC system (Shimadzu, Japan) coupled to a Q-TOF Maxis Impact mass spectrometer (Bruker Daltonics, Billerica, United States) equipped with an electrospray source. Separation was achieved on a SeQuant ZIC-pHILIC column (150 × 2.1 mm, 5 μm, Merck) at 0.175 ml/min. The column was kept at 95% of organic phase (acetonitrile) and at 45°C or 35°C for either positive or negative mode. The elution was performed by the gradient of aqueous phase (50 mM ammonium formate pH 3.5 in water for positive mode and 20 mM ammonium carbonate pH 9.2 in water for negative mode). Mass spectra were acquired at 0.67 Hz in the range of 30–1200 m/z in data dependent analysis. The amino acids were identified according to their exact masses (tolerance of 10 ppm), fragmentation pattern, and retention time of the corresponding pure standard mix. The mass spectra were analyzed with the MZmine2 software (Pluskal et al., 2010). Statistical analysis was performed with MetaboAnalyst 5.0 (Xia et al., 2009; Pang et al., 2021), and Prism 8.0 (GraphPad Software, San Diego, United States) was used to plot the data.

### Statistical Analyses

Student’s *t*-test was used for comparison between two conditions, and one-way ANOVA followed by Tukey’s test or two-way ANOVA followed by Sidak’s post-test for more than two conditions. Differences in survival curves were analyzed using the Log-Rank test, and egg hatching rates were compared by the Chi-square (χ^2^) test. In qPCR experiments, the ΔΔCq values, calculated from Cq values, were used for statistical analyses. Results were obtained from at least three independent determinations, and differences were considered significant at *p <* 0.05. Statistical analyses were performed using the Prism 8.0 software.

## Results

**FIGURE 1:**
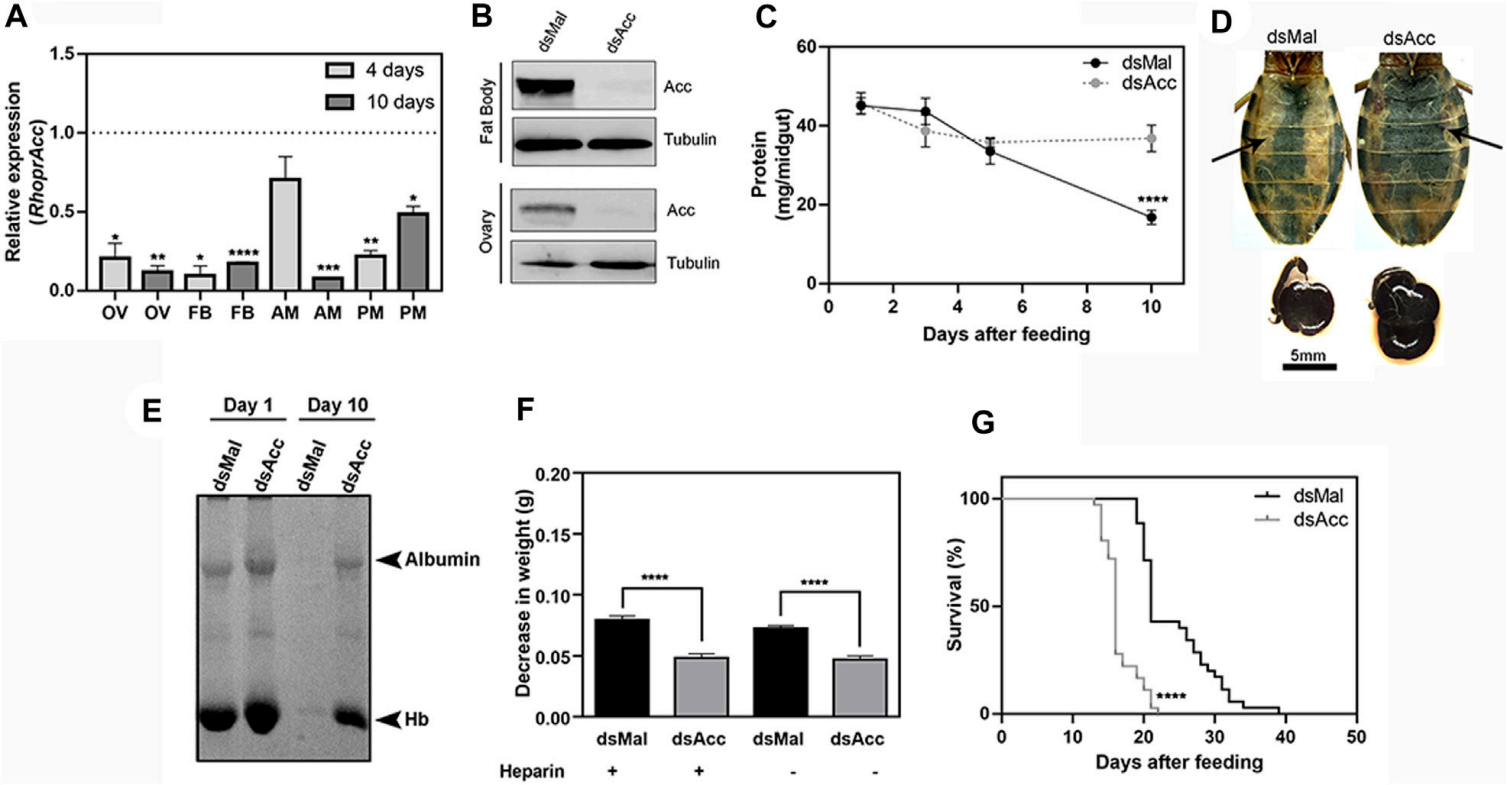
*Acc* knockdown in adult females compromised digestion and lifespan. Starving adult females were injected with 1 µg of dsRNA for *Acc* or *Mal* (control) and were fed 3 days later **(A)** Four and 10 days after blood meal, insects were dissected, and *Acc* RNA levels were determined by qPCR using *Rhopr18S* as a reference gene in ovaries (OV), fat bodies (FB), anterior midguts (AM), and posterior midguts (PM). Expression levels are relative to those from control insects (dashed line). Bars are means ± SEM for n = 3–5. (*), (**), (***), and (****): *p <* 0.05, 0.01, 0.001, and 0.0001, respectively, by Student’s *t*-test **(B)** Immunoblottings for ACC identification in fat bodies and ovaries’ homogenates (60–70 µg protein), obtained at day four after blood meal, representative images (n = 4) **(C)** At one, three, five, and 10 days after blood meal, insects were dissected and protein content was determined in total midguts. (****): *p* < 0.0001 by two-way ANOVA followed by Sidak’s post-test. Results are means ± SEM, n = 6. **(D)** Abdomens and midguts obtained from control and *AccKD* females, at 10 days after feeding. Arrows indicate the midguts. **(E)** The injected females were dissected at one and 10 days after blood meal, and the midgut protein content was analyzed by SDS-PAGE (12.5% polyacrylamide). Albumin and hemoglobin (Hb) bands are indicated. **(F)** Three days after dsRNA injection, insects were artificially fed on blood supplemented or not (control) with heparin. Females were weighed at days one and ten, and the results are expressed as the observed decrease in weight during this time. (****): *p* < 0.0001 by one-way ANOVA followed by Tukey’s test, n = 9–15. **(G)** After blood meal, the insect mortality was daily recorded. (****): *p <* 0.0001 by Log-Rank test, *n* = 27–28.

In order to evaluate how important *de novo* lipogenesis is for *R. prolixus* physiology, females were injected with dsAcc to inhibit *Acc* gene expression (AccKD females). Insects were dissected at days four and ten after blood meal to confirm gene knockdown (Figure 1A) and, at day four, when insects are metabolically very active, *Acc* gene expression was strongly decreased (78%–90% inhibition) in the fat body, ovary, and posterior midgut, whereas in the anterior midgut, it was not significantly affected. At day ten, *Acc* knockdown was achieved in all the analyzed organs, although in variable levels, confirming that gene silencing was persistent during the digestive cycle. The ACC protein presence was also investigated in the fat body and ovary at the fourth day after blood meal by immunoblots (Figure 1B), and it was dramatically decreased in dsAcc-injected females, confirming that *Acc* knockdown was very effective. Next, phenotypes related to digestion, lipid synthesis, and reproduction were evaluated.

Midgut total protein content was determined at different days after blood meal as a way to follow the digestion rate (Figure 1C), and although no difference was observed during the first 5 days, after this time, digestion was compromised in the AccKD females since midgut total protein amount did not further decrease as in the control ones. This was confirmed by the visual observation of the midgut (Figure 1D), and by analysis of protein composition by SDS-PAGE, where much larger amounts of remaining blood proteins (mostly albumin and hemoglobin) were present in the midgut of AccKD females 10 days after feeding (Figure 1E). We noticed that the midguts from the AccKD insects had reduced peristaltic activity (data not shown) and were more fragile, and their contents seemed harder, suggesting that some coagulation might have occurred. To check this possibility, insects were artificially fed on blood enriched with heparin, and the decrease in insect weight between days one and ten after blood meal was determined. No difference was observed, and the AccKD insects continued to show the same arrest in digestion when compared to control insects (Figure 1F). In this experiment, insect weight loss that occurred between one and 10 days after feeding was used to estimate digestion progress. The AccKD females also had a shorter lifespan, with median survival of 16 days versus 21 days in the control group (Figure 1G).

**FIGURE 2:**
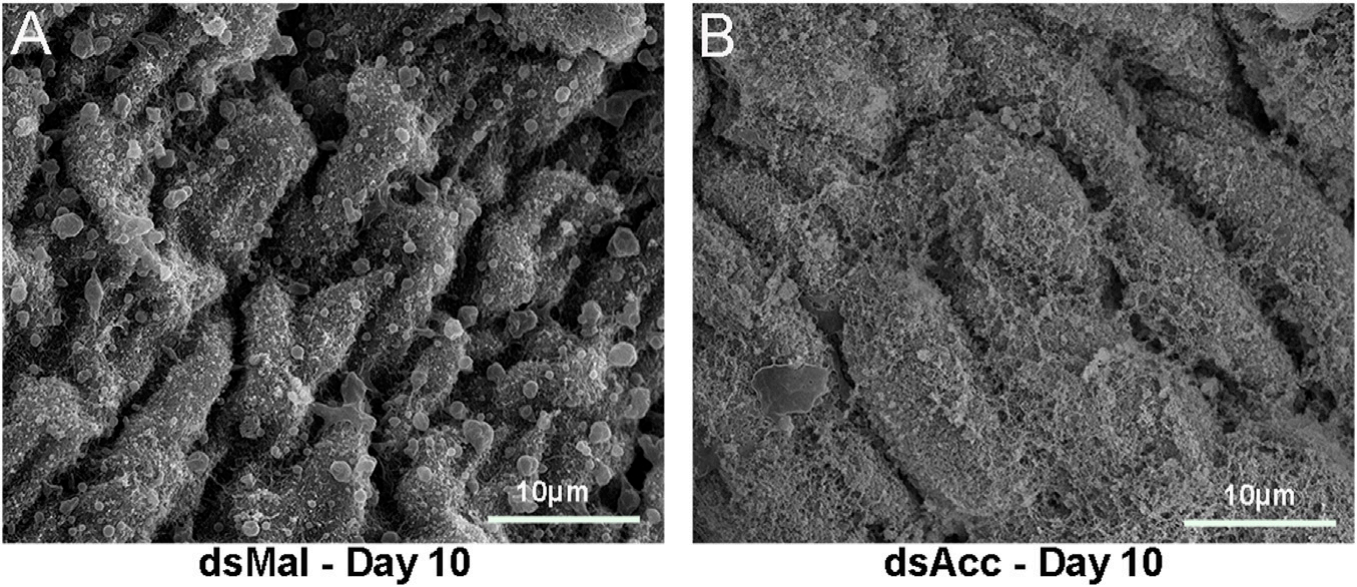
*Acc* knockdown affected perimicrovillar membranes. Starving adult females were injected with 1 µg of dsRNA for *Acc* or *Mal* (control) and were fed 3 days later. Insects were dissected at 10 days after blood meal, and the midguts were collected, washed, and processed for scanning electron microscopy. **(A)** dsMal; **(B)** dsAcc.

In most insects, the midgut synthesizes a peritrophic matrix, a chitin-based matrix that surrounds the food bolus (Bolognesi et al., 2008), but in hemipterans, instead, the midgut produces an extra-cellular membrane network, the perimicrovillar membrane (PMM), which covers the microvilli and is synthesized after feeding (Billingsley and Downe, 1986; Damasceno-Sá et al., 2007). As in AccKD females digestion was impaired, and as the PMM consists of true phospholipidic bilayer membranes, we considered it could be affected and thus examined the midgut luminal surface by scanning electron microscopy (Figure 2). At 10 days after blood meal, the anterior midgut epithelium from AccKD females had a different surface appearance when compared to the control midgut. In control females, a membranous meshwork and membrane blebs were observed but, in the midgut of the AccKD insects, the membranes appeared disorganized, with a pattern that seemed in accordance with the presence of more abundant membranes, as previously reported (Damasceno-Sá et al., 2007).

**FIGURE 3:**
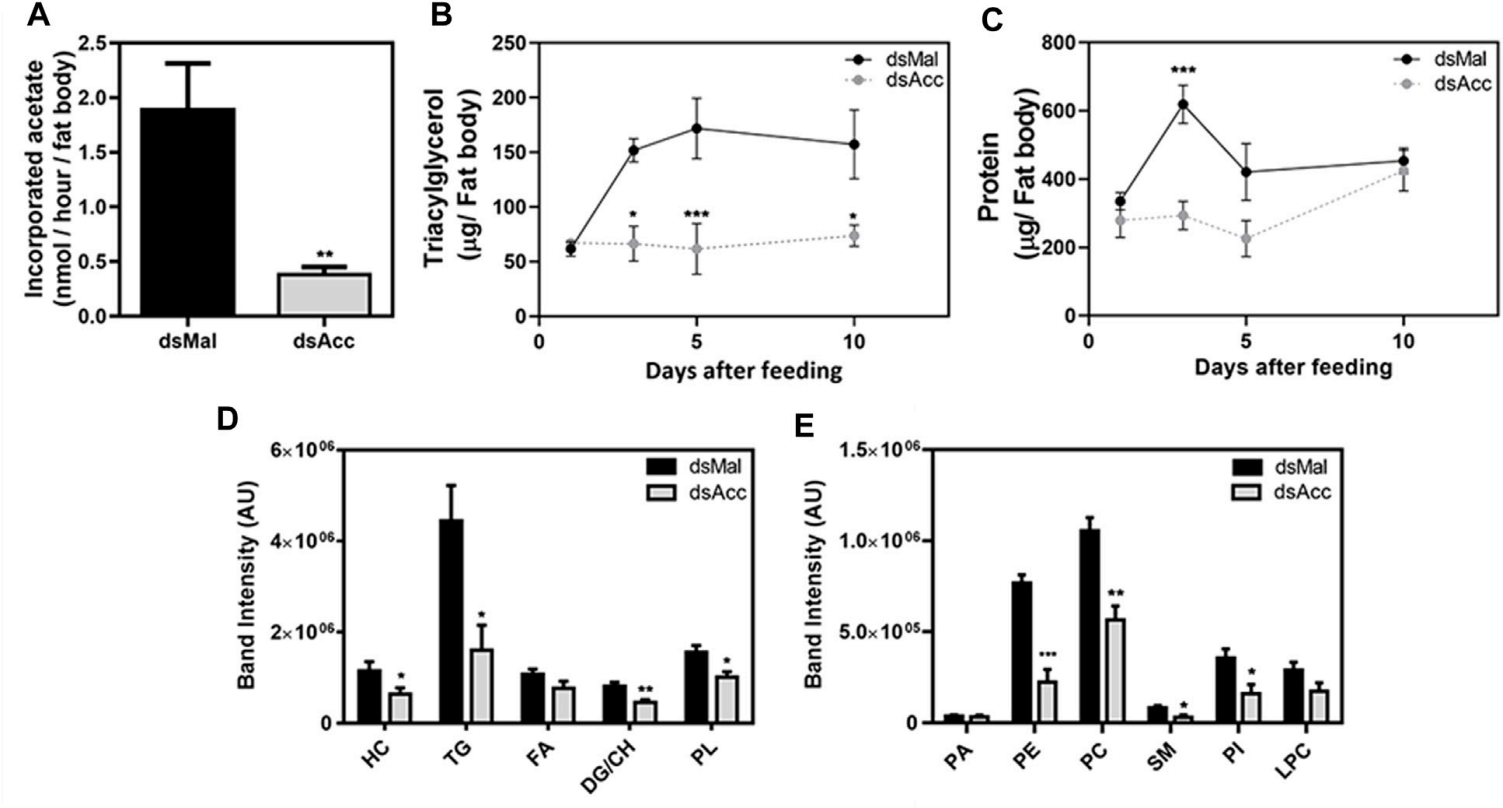
Decreased lipid synthesis and accumulation in the fat body upon *Acc* knockdown. Starving adult females were injected with 1 µg of dsRNA for *Acc* or *Mal* (control) and were fed 3 days later. **(A)** Three days after blood meal, insects were dissected and the isolated fat bodies were incubated in the presence of ^3^H-acetate for 1h for the determination of its incorporation into lipids. Bars are means ± SEM (n = 7). At 1, 3, 5, and 10 days after blood meal, insects were dissected and fat bodies were collected for **(B)** triacylglycerol and **(C)** protein content determination. The results are means ± SEM for five determinations. Alternatively, insects were dissected at day five after blood meal, the fat bodies were collected, and **(D)** the neutral lipid and **(E)** phospholipid composition was examined by TLC, followed by densitometry. Bars are means ± SEM for 7 (neutral lipids) or 5-6 (phospholipids) determinations. (*), (**), and (***): *p <* 0.05, 0.01, and 0.001, respectively, by Student’s *t* test **(A,D,E)** or two-way ANOVA followed by Sidak’s post-test **(B,C)**. HC: hydrocarbon; TG: triacylglycerol; FA: fatty acid; DG: diacylglycerol; CH: cholesterol; PL: phospholipids; PA, phosphatidic acid; PE, phosphatidylethanolamine; PC, phosphatidylcholine; SM, sphingomyelin; PI, phosphatidylinositol; LPC: lysophosphatidylcholine.

**FIGURE 4:**
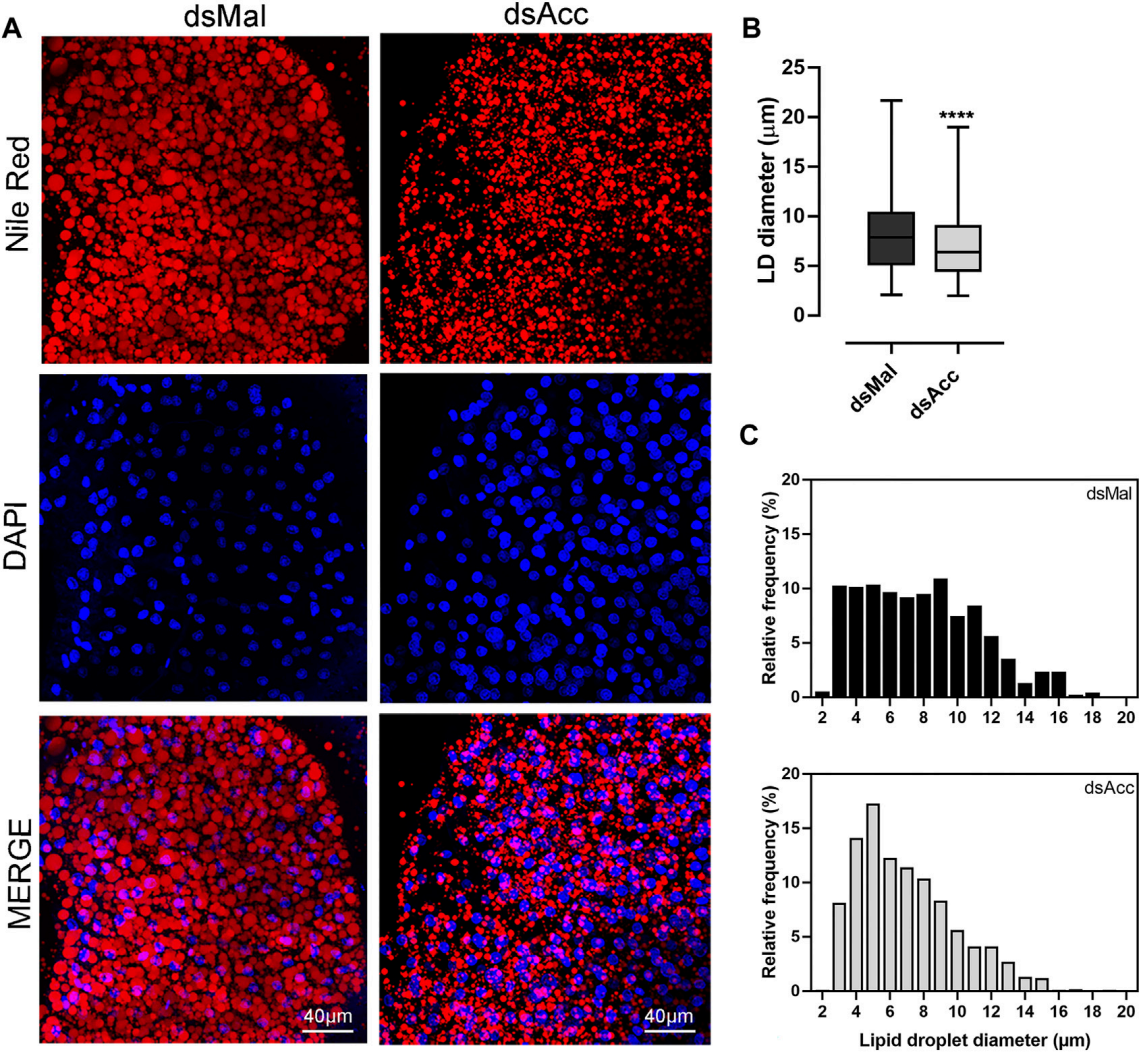
Smaller lipid droplets in the fat body of *Acc* knockdown females. Starving adult females were injected with 1 µg of dsRNA for *Acc* or *Mal* (control) and were fed 3 days later. **(A)** At the fifth day after blood meal, freshly dissected fat bodies were stained with Nile Red and DAPI and observed under a laser scanning confocal microscope. Bars: 40 μm. **(B)** Maximum diameters of lipid droplets (LDs) were determined from four to five images obtained from at least four insects, in three experiments, and the results are means ± SD for at least 1037 LDs per group. (****): *p <* 0.0001 by Student’s *t*-test. **(C)** Diameter distribution histograms of the LDs from panel **(B)**

To test the inhibition of *de novo* lipogenesis after dsAcc injection, fat bodies were incubated in the presence of ^3^H-acetate, and the lipid synthesis activity was determined. A decrease of 80% in incorporation of acetate into lipids was observed in AccKD females when compared to control, confirming the effectiveness of *Acc* silencing (Figure 3A). As a consequence, the fat body of the AccKD females did not accumulate lipids after the blood meal. The fat body TG content remained similar to the unfed condition, in contrast to the control females, where the fat body TG content increased after feeding (Figure 3B). The fat body lipid composition was analyzed, and among neutral lipids, not only TG but also hydrocarbons and diacylglycerol were present in much smaller amounts (Figure 3D). The phospholipid content was also reduced, and significant decreases were observed not only in phosphatidylethanolamine and phosphatidylcholine, the most abundant ones, but in other classes, as well (Figure 3D,E). The inhibition of *Acc* gene expression also resulted in smaller fat bodies in a more general way, with reduced protein contents (Figure 3C). In insects, as in other organisms, lipids are stored in the fat body in lipid droplets (Arrese and Soulages, 2010; Toprak et al., 2020), and thus these organelles were examined by confocal fluorescence microscopy. Lipid droplets from the AccKD females were smaller in average when compared to the control ones (Figure 4A,B). The analysis of lipid droplet population according to diameter distribution showed that the AccKD insects had a larger proportion of those with smaller diameters, between 3 and 8 µm (∼73% versus ∼ 58% in the control group) (Figure 4C).

**FIGURE 5:**
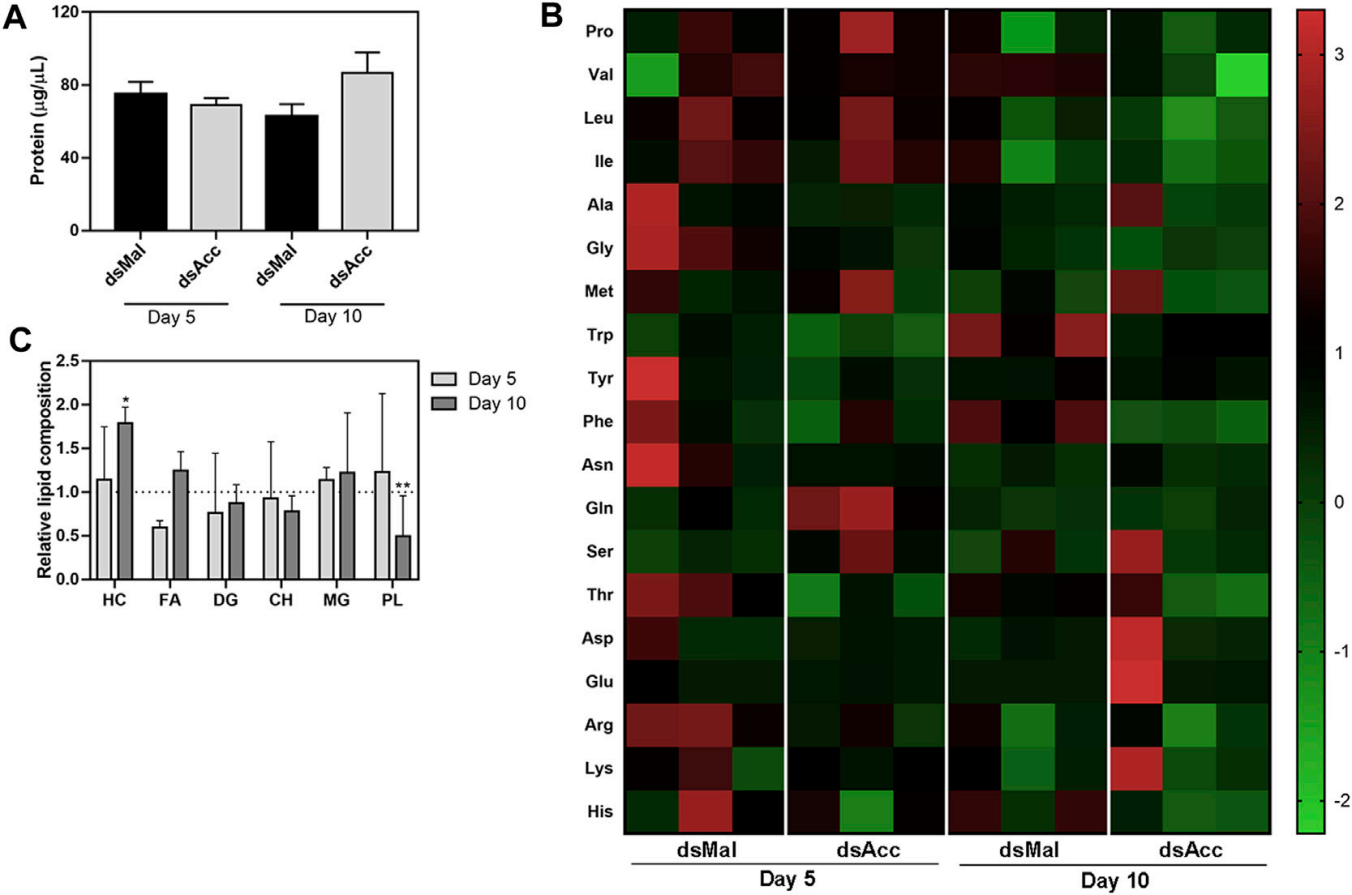
Hemolymph protein, amino acid, and lipid composition after *Acc* knockdown. Starving adult females were injected with 1 µg of dsRNA for *Acc* or *Mal* (control) and were fed 3 days later. At days five and ten after blood meal, hemolymph was collected for the determination of **(A)** the total protein content (means ± SEM, n = 8) and **(B)** the amino acid composition (data shown for three individual samples in each group). **(C)** The hemolymph lipid composition was also evaluated at the same days, and the results are expressed relative to control values for each lipid class (dashed line). Results are means ± SEM for six determinations. (*) and (**): *p <* 0.05 and 0.01, respectively, by Student’s *t*-test when compared to the corresponding control.

In spite of the compromised blood digestion, the total protein concentration in the hemolymph was not affected in the AccKD females, at either five or 10 days after feeding (Figure 5A). Because amino acids released by the digestion of ingested blood proteins are a major substrate for lipid synthesis in this insect (Saraiva et al., 2021), and as the digestive process was compromised in the AccKD females, hemolymphatic amino acid abundance was evaluated. The inhibition of *Acc* expression did not significantly alter the hemolymph amino acid composition (Figure 5B), demonstrating that, although lipid amount was dramatically decreased in the fat body, this was not due to substrate shortage. Note that, in this analysis, the abundance of each amino acid type is compared between the tested conditions (the results for three individual insects per group are shown), but it is not possible to compare relative concentrations of the different amino acids’ species in the same hemolymph sample. Unexpectedly, at the fifth day after blood meal, the hemolymph lipid composition was not significantly altered and, at day ten, only an increase in hydrocarbon amount (∼78%) and a decrease in phospholipids (∼50%) were observed (Figure 5C).

**FIGURE 6:**
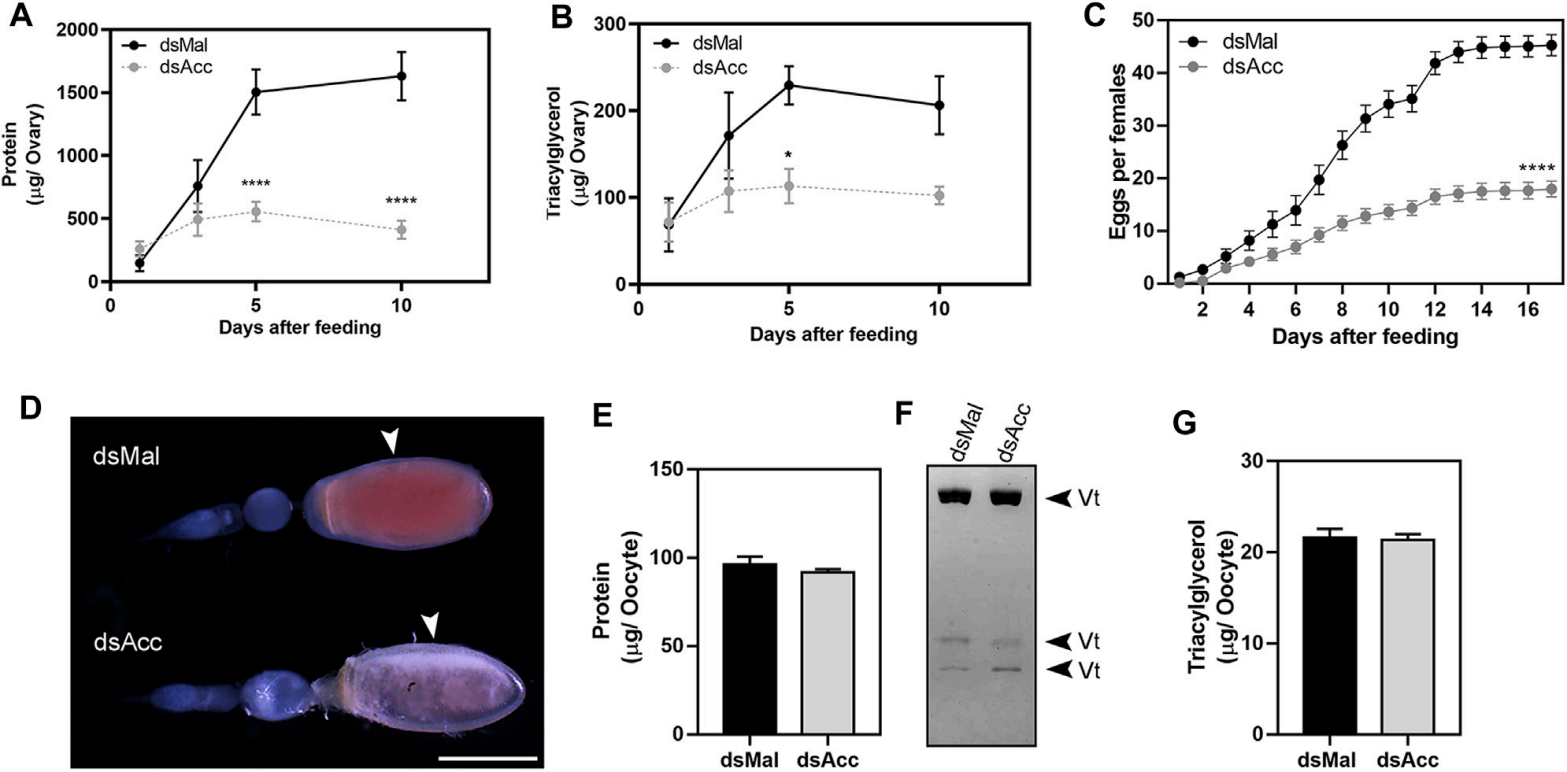
*Acc* knockdown impaired reproduction. Starving adult females were injected with 1 µg of dsRNA for *Acc* or *Mal* (control) and were fed 3 days later. At days one, three, five, and ten after blood meal, the insects were dissected, the ovaries were collected, and total contents of **(A)** proteins and **(B)** triacylglycerol were determined (n = 6). **(C)** After feeding, the cumulative oviposition was recorded (n = 35). (*) and (****): *p <* 0.05 and 0.0001, respectively, by two-way ANOVA followed by Sidak’s post-test. **(D)** Ovarioles dissected at day 12 after blood meal, from dsMal and dsAcc injected females, examined under a stereomicroscope. The arrowheads indicate the chorionated oocytes. Bar: 1.0 mm. **(E)** Total protein content in individual chorionated oocytes, dissected 11–13 days after feeding (means ± SEM; n = 4). **(F)** Analysis of proteins present in chorionated oocytes by SDS-PAGE (7.5% polyacrylamide). Vt: vitellin. **(G)** Triacylglycerol content in individual chorionated oocytes (means ± SEM; n = 9).

**FIGURE 7:**
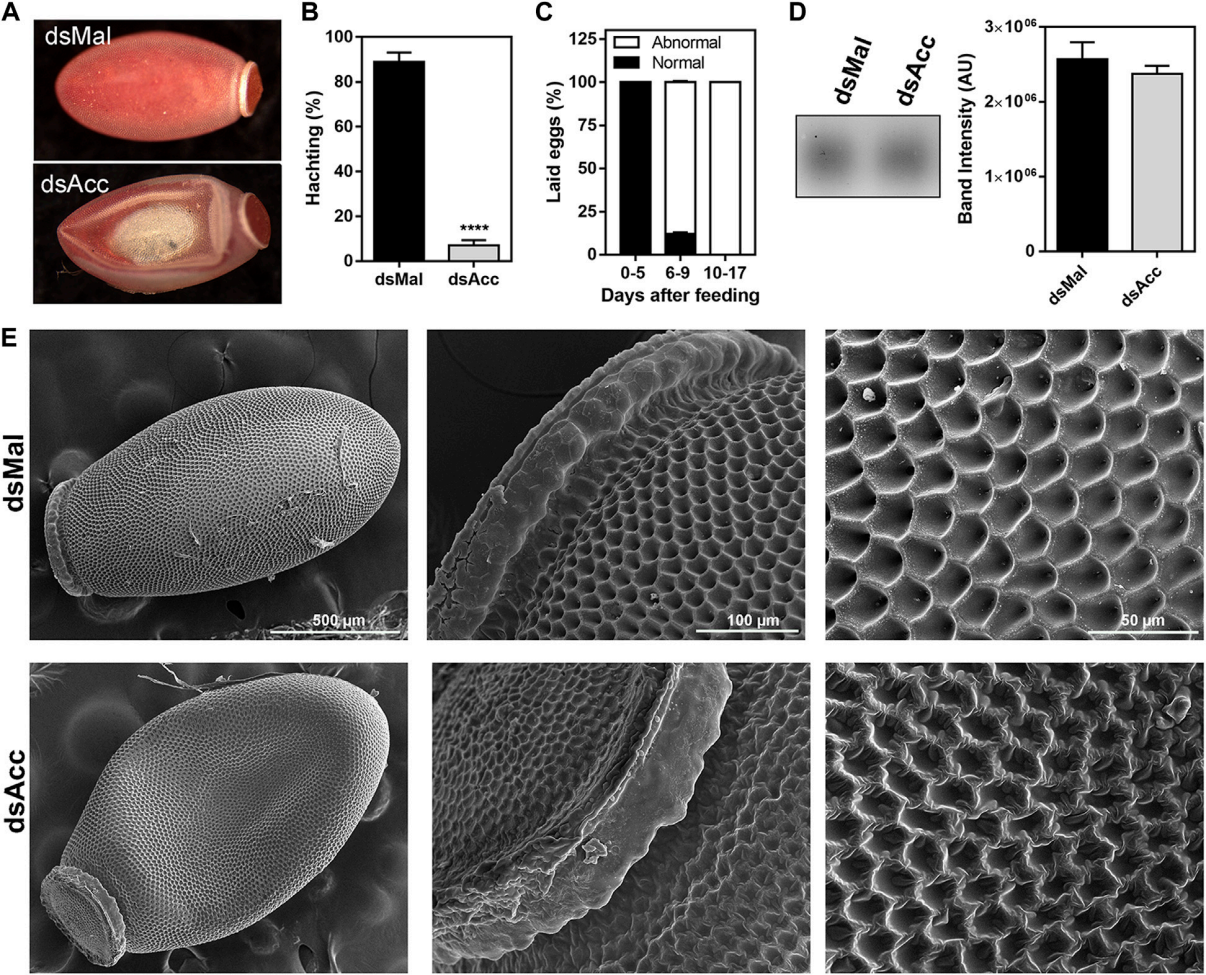
*Acc* knockdown females deposited eggs with defective chorion structure. The starving adult females were injected with 1 µg of dsRNA for *Acc* or *Mal* (control) and were fed 3 days later. **(A)** Chorionated eggs, deposited by dsMal and dsAcc injected females. Eggs were collected on different days of the oviposition cycle, and **(B)** the total number of hatched eggs in each group was determined. (****): *p* < 0.0001, by χ2 test, n = 1687. **(C)** Frequency of morphological alterations among eggs laid by females injected with dsAcc (n = 167). **(D)** Hydrocarbons extracted from the eggshells of eggs laid by AccKD and control females were evaluated by TLC, followed by densitometry (n = 3). **(E)** The laid eggs were processed for scanning electron microscopy, and their surface ultrastructure was examined.

After feeding, the ovaries of the AccKD females did not develop as the control ones, as could be observed by the total protein and TG contents in the following days after blood meal (Figure 6A,B). Thus, these females had a 60% decrease in egg laying when compared to the dsMal injected ones (Figure 6C). The oocytes dissected from the AccKD females were visually unlike the control (Figure 6D), but there was no difference in chorionated (mature) oocyte protein and TG contents or in protein composition (Figure 6E–G). The same result for the TG and protein content was obtained for laid eggs (data not shown). Nonetheless, the eggs from the AccKD females showed a collapsed phenotype after oviposition and a very low hatching rate (Figure 7A–C).

Because of this egg phenotype, which could indicate a dehydration condition, we questioned whether *Acc* silencing affected the hydrocarbon content on the egg surface. Thus, the eggs were emptied of their contents and the eggshells were subjected to lipid extraction with hexane in order to evaluate the surface hydrocarbon content, but no difference was observed (Figure 7D). On the other hand, the examination of the eggshell surface by scanning electron microscopy revealed that it was damaged in the eggs produced by the AccKD insects (Figure 7E), thus demonstrating that the inhibition of *Acc* gene expression altered the chorion (eggshell) synthesis and final ultrastructural pattern.

## Discussion

**FIGURE 8:**
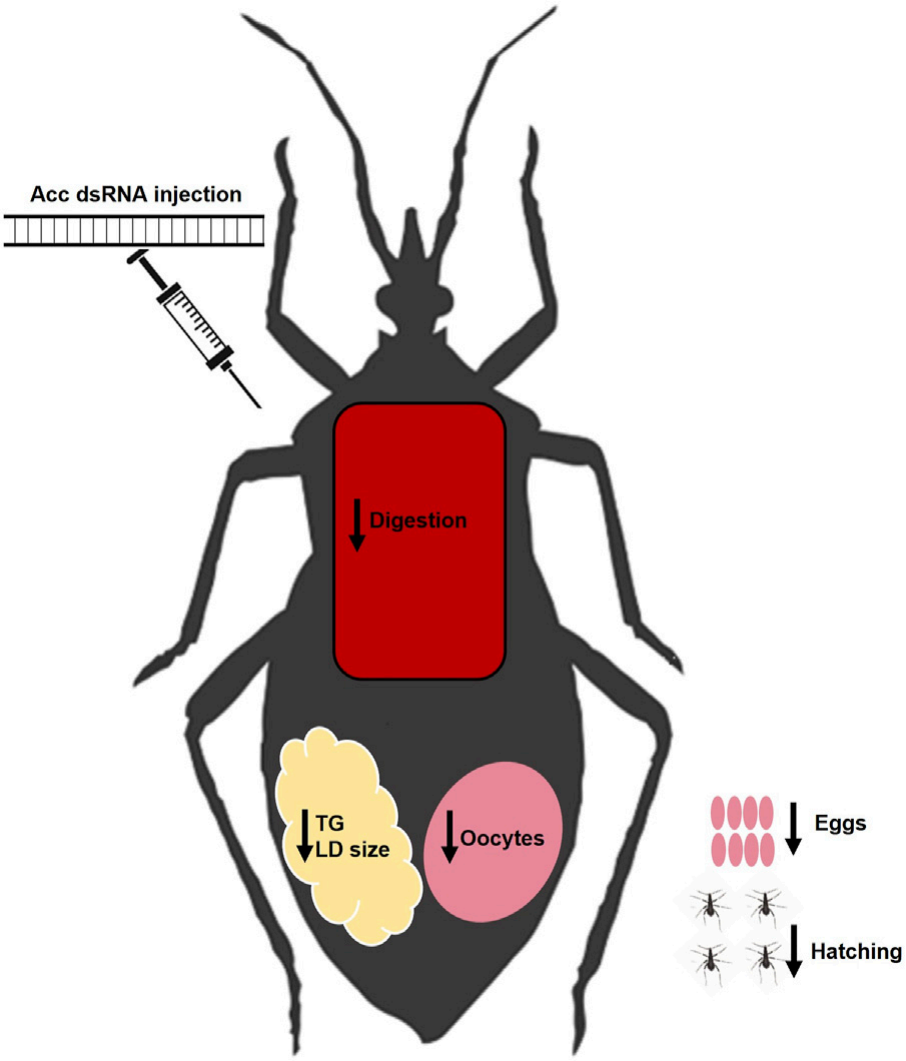
Summary of effects of *Acc* knockdown in adult females of *R. prolixus.* After the inhibition of *Acc* gene expression by the injection of dsRNA, the fat bodies of the AccKD females synthesized and stored lower amounts of lipids, and had smaller lipid droplets. These females laid fewer eggs, which did not hatch and had chorion defects. Although digestion was disturbed, concentrations of amino acids and proteins in the hemolymph were not affected. LD: lipid droplet; TG: triacylglycerol.

In this study, we were able to systemically inhibit *Acc* gene expression in adult females of *R. prolixus* to address the participation of ACC activity in insect lipid metabolism and reproduction, and the main results are summarized in Figure 8. The effect of *Acc* knockdown in digestion was very clear and easily noticed, and different from the previously described in *A. aegypti*, in which ACC silencing did not affect digestion after a blood meal but, instead, FASN silencing did (Alabaster et al., 2011). Despite these two enzymes take part of the same pathway for fatty acids synthesis, it is not possible to say whether the effects observed in the digestive process in *R. prolixus* and in the mosquito are related. It is noteworthy the effect of *Acc* gene expression inhibition on the PMM phenotype. The midgut surface of AccKD females appeared to be more abundantly coated by the PMM, according to previous descriptions of the midgut surface ultrastructure, which in hemipterans shows an increase in PMM abundance after feeding (Billingsley and Downe, 1986; Damasceno-Sá et al., 2007). This was an unexpected result, as lipid synthesis was inhibited in AccKD females. On the other hand, digestion was also arrested, and this could contribute to diminished midgut traffic and thus reduced PMM degradation and elimination in the feces. Nevertheless, although membranes seemed more abundant, it is not possible to conclude that this was actually the case, and more detailed analyses are required to verify this possibility. The AccKD females had a decreased lifespan, and although the arrest in digestion may have strongly contributed to this phenotype, it is not possible to identify all the factors that might be involved. In *Triatoma infestans*, the chemical inhibition of cuticular lipid synthesis resulted in an increase in mortality after exposure to insecticide (Juárez, 1994a).

Importantly, the observation that protein and amino acid concentrations in the hemolymph after *Acc* silencing were not reduced, even though digestion was disturbed, demonstrated that the effects on the fat body lipid composition and oogenesis were not due to a global lack of substrates. It is possible that the absorption of nutrients by the midgut was altered, but, if this occurred, it did not affect protein or amino acid abundance in the hemolymph.

The decrease in the *de novo* synthesis of lipids by the fat body in the AccKD females resulted not only in a smaller TG reservoir in this organ but also in a general decrease in the amount of lipids of the various classes, including phospholipids, and showed that the ACC activity is crucial to rebuild lipid contents during the digestive/reproductive cycle. Nonetheless, differences in the lipid composition of the fat body, hemolymph, and ovaries between AccKD and control females seemed not as big as the observed decrease in the lipid synthesis activity, indicating that part of the lipids present in the organs are not derived from *de novo* lipogenesis but are directly absorbed from the ingested blood, as previously described in *R. prolixus* and other insects (Grillo et al., 2007; Gondim et al., 2018).

In accordance with the lower TG content in the fat bodies of the AccKD females, lipid droplets were smaller than in control insects, as they did not grow as expected after the blood meal due to limited lipid synthesis. The same phenotype was also observed in females silenced for an insulin receptor, which regulates lipid synthesis by *R. prolixus* fat body, and silenced for Gpat1, the enzyme that catalyzes the first step in the TG synthesis pathway (Alves-Bezerra et al., 2017; Silva-Oliveira et al., 2021). On the other hand, when lipophagy or mitochondrial *β*-oxidation were inhibited in this insect during starvation, through the knockdown of *Atg6* or *Atg8*, or of mitochondrial trifunctional protein A subunit, lipid droplets were larger than the control ones, as they were not mobilized in the same way (Santos-Araujo et al., 2020; Arêdes et al., 2022). These results confirmed that, in the fat body cells of triatomines, lipid droplets are dynamic organelles that undergo changes in volume during the feeding cycle according to the nutritional condition, as known for other models, as *Drosophila* and mammalian cells (Guo et al., 2008; Walther and Farese, 2012).

Notwithstanding the reduced lipid content in the fat body of the AccKD females, the hemolymph lipid composition was not deeply affected. Whereas at the fifth day after feeding, no difference was observed, at the 10th day, only hydrocarbon and phospholipid contents differed from the control. In the same way, in the insects mentioned above, silenced for the insulin receptor or for Gpat1, which also had a deficiency in fat body lipid synthesis, the hemolymph lipid content was not affected (Alves-Bezerra et al., 2017; Silva-Oliveira et al., 2021). Thus, the hemolymph lipid composition seems to be under a strict control, and it is not easily affected, not being a good readout for modifications in the insect lipid metabolism or reservoirs. However, the possibility that the inhibition of lipid synthesis in the AccKD females resulted in specific modifications in the lipid species composition that somehow affected the Lp function cannot be discarded, and this would require further investigation.

Although proteins and lipids were present in the hemolymph, the ovaries of the AccKD females did not develop normally during vitellogenesis and were much smaller than in control females, and there was a decrease of around 60% in the number of laid eggs. Surprisingly, chorionated oocytes and eggs from both AccKD and control females had the same contents of protein and lipids, but only a very small proportion of the eggs deposited by the ACC-deficient females hatched. Thus, it seems that the eggs laid by the AccKD females had some other problem that was not a shortage of yolk components, and it was possibly related to the chorion disarranged structure. Similar effects were observed in *A. aegypti* females that were silenced for *Acc*. They also laid fewer eggs than the control insects, and they were not viable, showing defects in the eggshell, which was more porous (Alabaster et al., 2011). The insect eggshell is produced at the end of vitellogenesis by the follicular epithelium cells, which at this time synthesize and secrete chorion proteins (Paul et al., 1972; Medeiros et al., 2011). The eggshell also contains lipids, which act to prevent desiccation, among other possible functions. The *D. melanogaster* eggshell contains an inner wax layer that was shown to derive from lipid vesicles secreted by the follicle cells during choriogenesis (Papassideri et al., 1993; Cavaliere et al., 2008). Similarly, in *T. infestans*, the eggs *de novo* synthesize hydrocarbons, besides incorporating them from the hemolymph, and the inhibition of hydrocarbon synthesis in the eggs resulted in decreased wax deposition on the egg surface and lower hatchability (Juárez, 1994b). Hence, the inhibition of *Acc* gene expression might disturb the production of eggshell lipids, proteins, or both, affecting the eggshell structure and contributing to the reproductive failure in these insects. However, no difference was observed in the total hydrocarbon amount extracted from eggshells obtained from AccKD females, although specific effects on the hydrocarbon specimen composition or changes in the contents of other lipid classes that may have occurred after *Acc* knockdown cannot be ruled out, and this issue deserves further investigation.

It is noteworthy that the knockdown of *Acc* resulted in multiple phenotypes, as described here, but not all of them clearly related to a deficiency in lipid synthesis. It is possible that decreasing ACC activity, which has acetyl-CoA as a substrate, alters the concentration and/or cellular distribution of this metabolite. Acetyl-CoA is a metabolic hub due to its intersection with various metabolic pathways. Moreover, it is the acetyl donor for protein acetylation, taking part not only in the control of gene expression through histone acetylation but also in the direct regulation of metabolic events due to the acetylation of innumerous non-histone proteins (Shi and Tu, 2015; Xia et al., 2020). Thus, it is possible that diverse cellular processes may somehow be disturbed by this supposed unbalance in acetyl-CoA availability. Another point that deserves attention is a possible modification of fatty acid composition due to *Acc* silencing. In insects, as in mammals, fatty acids have a key role in the regulation of cellular metabolism as they are ligands of diverse transcription factors/nuclear receptors (Majerowicz and Gondim, 2013), and then, alterations in the fatty acid composition might possibly affect various unpredicted events.

Lipid metabolism has been deeply studied in the Chagas disease vector *R. prolixus*, and here we have investigated the contribution of the ACC activity to preserve the insect’s biological functions. This study unravels a central role for *de novo* lipogenesis in the buildup of lipid stores and reproduction in this insect, being important to face the metabolic challenge that hematophagy represents.

## Abbreviations

Acc, acetyl-CoA carboxylase; AccKD females, Acc knockdown females; ESI, electrospray source; FASN, fatty acid synthase; Lp, lipophorin; LD, lipid droplet; PBS, phosphate buffered saline; PMM, perimicrovillar membrane; TG, triacylglycerol.

## Data Availability

The raw data supporting the conclusions of this article will be made available by the authors, without undue reservation.
