# Supplementary material for: Deficiency of Acetyl-CoA Carboxylase Impairs Digestion, Lipid Synthesis, and Reproduction in the Kissing Bug Rhodnius prolixus
Source: Front Physiol. 2022 Jul 22;13:934667. doi: 10.3389/fphys.2022.934667 (PMC9353303; doi:10.3389/fphys.2022.934667)
Supplement: Supplementary file 1 [file DataSheet1.PDF]

Supplementary data

Supplementary Table 1: Primer sequences used in the present study.

Primer	Sequence (5' - 3')
**Primers used in qPCR reactions**	
RhoprAcc_F	TGGGCTGGAACCGTAGTTGCG
RhoprAcc_R	TGCGGGATCGGCTGGAAGTTGT
Rhopr18S_F	TCGGCCAACAAAAGTACACA
Rhopr18S_R	TGTCGGTGTAACTGGCATGT
**Primers used for dsRNA synthesis**	
RhoprAcc_F	TAATACGACTCACTATAGGGAG CCTAATAGGCAGAAGCTAGG
RhoprAcc_R	TAATACGACTCACTATAGGGAG CGTGACTTCAGTTCATCCAT
T7 minimal	TAATACGACTCACTATAGG

F, forward
R, reverse
